# Reported prevalence of interprofessional conflict in Indian health care, training adequacy and patient safety consequences: a multi-state cross-sectional study

**DOI:** 10.3389/frhs.2026.1852139

**Published:** 2026-08-25

**Authors:** Amrita Sarkar, Sonal Dayama, Meet Chauhan, Medha Mathur, Mamta Gehlawat, Debjit Roy

**Affiliations:** 1 Department of Community Medicine, Tomo Riba Institute of Health and Medical Sciences (TRIHMS), Naharlagun, Arunachal Pradesh, India; 2 Department of Community Medicine, ESIC Medical College, Hyderabad, Telangana, India; 3 Department of Community Medicine, Shantabaa Medical College, Amreli, Gujarat, India; 4 Department of Community Medicine, Geetanjali Medical College & Hospital, Geetanjali University, Udaipur, Rajasthan, India; 5 Department of Community Medicine, Government Medical College, Mahabubabad, Telangana, India; 6 Department of Psychiatry, IQ City Medical College, Durgapur, West Bengal, India

**Keywords:** conflict, health professional, patient safety, training, workplace stress

## Abstract

**Background:**

Interprofessional conflict can compromise, teamwork, workforce stability, and patient safety particularly in overstretched public health systems in India. Although international literature highlights this issue, multi-state evidence from India involving different healthcare cadres remains limited.

**Methods:**

A cross-sectional survey was conducted among healthcare professionals from Arunachal Pradesh, Rajasthan, Gujarat, and Telangana Data were collected using a validated 12-item questionnaire on a 5-point Likert scale. The survey assessed the prevalence of interprofessional conflict, its perceived impact on patient care and teamwork, and the gap between the adequacy and importance of conflict-management training. Role-based differences were examined using ANOVA, and associations were explored with Spearman's correlation.

**Results:**

Mean age of 566 health care professionals (doctors (52.7%), nurses (35.7%), and interns (11.6%)) surveyed was 29.8 years. Interprofessional conflict was commonly reported (mean 3.70; 65% agreement). Participants indicated that conflict affected care quality (mean=3.60, SD = 1.23), increased workplace stress (mean=3.51,SD = 1.15), and hindered collaboration (mea*n* = 3.32, SD = 1.12). A notable training gap emerged between perceived adequacy (2.95) and importance (3.88), with a difference of 0.93 (Cohen's d = 0.78, *p* < 0.001). Nurses reported higher stress levels (F = 6.84, *p* = 0.001), while interns perceived lower training adequacy (F = 9.97, *p* < 0.001). Conflict scores showed moderate correlations with reported delays or errors in care (*ρ* = 0.43–0.69).

**Conclusions:**

Interprofessional conflict is common and serious in India's healthcare settings; nearly two-thirds of participants report that it occurs. The findings show this conflict is a systemic issue that compromises care quality, increases workplace stress, and weakens team collaboration. Moderate correlations between conflict and care delays or errors highlight its risks to patient safety. The study urges making structured, cadre-sensitive interprofessional conflict-management training a top systems-level priority to strengthen teamwork, workforce well-being, and patient outcomes in India's public health system.

## 1 Introduction

Interprofessional conflict, broadly defined as tension, disagreement, or breakdown in collegial working relations among members of a healthcare team, constitutes a well-documented threat to patient safety, workforce wellbeing, and organisational effectiveness in health systems worldwide (1). The World Health Organization (WHO) has identified communication failure and team dysfunction as root causes in a substantial proportion of sentinel events and preventable adverse outcomes globally, with unresolved interprofessional conflict recognised as a primary driver of both (2). A consequence of interprofessional conflict is the erosion of collaborative practice. In one survey conducted in acute care settings, Hendel et al. reported that nurses, physicians, and allied health professionals spent a substantial proportion of their working time managing interpersonal tensions, highlighting the potential impact of conflict on collaborative practice and patient care (3).

Effective interprofessional collaboration is widely recognised as a cornerstone of safe, high-quality, and patient-centred healthcare. Evidence indicates that unresolved conflict among healthcare professionals can impair communication, reduce team effectiveness, contribute to burnout and job dissatisfaction, and compromise patient safety and quality of care. These effects have important implications for health system performance and reinforce the need to strengthen interprofessional competencies among healthcare professionals. In this context, fostering collaborative practice aligns with broader global efforts to improve healthcare quality and workforce capacity, including the objectives of Sustainable Development Goal 3 (Good Health and Well-being) and Sustainable Development Goal 4 (Quality Education) (4).

In India, the convergence of high patient volumes, constrained human resources, and entrenched occupational hierarchies amplifies the consequences of interprofessional conflict within public health facilities. India continues to face challenges related to the recruitment and retention of healthcare professionals, particularly nurses and frontline health workers. Studies have shown that occupational stress, workplace toxicity, interpersonal conflict, and poor organizational support contribute to burnout and turnover intentions among healthcare workers, with potential implications for the quality and continuity of patient care (5, 6). Despite the growing global consensus on interprofessional education (IPE) as a mechanism for improving collaborative clinical practice, a principle reflected in the Competency-Based Medical Education curriculum for MBBS graduates of India (7) and revised nursing curriculum frameworks issued by the Indian Nursing Council (8), structured conflict management training remains largely absent from undergraduate and postgraduate health professions curricula in India (9). This omission is particularly consequential given the hierarchical organisation of Indian clinical teams, where power gradients between doctors, nurses, and support staff may suppress conflict disclosure among junior workers while simultaneously intensifying its psychological and clinical consequences (10).

Despite a growing body of international evidence on the consequences of interprofessional conflict for patient safety and workforce outcomes, quantitative multi-site evidence from the Indian healthcare context remains sparse. Existing Indian studies on workplace interprofessional relations are predominantly qualitative, single-institutional, or restricted to nursing cadres; few have simultaneously examined perceived conflict prevalence, its patient safety implications, and the adequacy of institutional training provisions within a unified analytic framework (11, 12). The absence of comparative multi-state data also precludes inference as to whether observed training deficits are systemic at a national level or are institution-specific phenomena amenable to localised interventions. Moreover, the perspectives of three clinical groups (doctors, nurses, and interns) are rarely integrated within a single study, leaving the role of professional hierarchy in shaping conflict perceptions and its consequences insufficiently characterised. The present study was designed to address these gaps by quantitatively assessing the perceived prevalence and consequences of interprofessional conflict, the adequacy of current institutional training provisions, and the correlation structure linking conflict exposure to clinical outcomes, across a geographically diverse, multi-state, multi-cadre sample of Indian healthcare professionals.

### 1.1 Objectives

To determine the reported perceived prevalence of interprofessional conflict among doctors, nurses, and interns employed in public and private sector healthcare facilities across Arunachal Pradesh, Rajasthan, Gujarat, and Telangana, and to assess the gap between current conflict management training and the perceived need for such training.

## 2 Methodology

### 2.1 Study design

A multi-state, institution-based, analytical cross-sectional study was conducted among healthcare professionals employed in public and private sector hospitals across four Indian states. The cross-sectional design was appropriate for estimating the point prevalence of attitudes, perceptions, and self-reported experiences at a defined time point, and for identifying associations between conflict-related constructs and professional characteristics without manipulation of the exposure. This report adheres to the Strengthening the Reporting of Observational Studies in Epidemiology (STROBE) checklist for cross-sectional studies (13). Data were collected during the period [1st January to 31st December 2024]; the data collection window within each facility did not exceed six weeks.

### 2.2 Study setting

The study was conducted across five secondary- and tertiary-care public and private sector hospitals in Arunachal Pradesh (public hospital in Northeast India), Rajasthan (private hospital in North-West India), Gujarat (private hospital in Western India), and Telangana (South India) One hospital in each state except Telangana, where two public hospitals were surveyed. These states were purposively selected to represent heterogeneity in health system infrastructure, healthcare worker density, geographic accessibility, and socioeconomic context, with the aim of enhancing the external validity of findings within the Indian healthcare sector landscape. The four states encompassed in the present study, Arunachal Pradesh, Rajasthan, Gujarat, and Telangana, collectively represent a purposively heterogeneous cross-section of India's socio-geographic, epidemiological, and health system landscape. Arunachal Pradesh, a predominantly tribal state in Northeast India characterised by sparse healthcare infrastructure and geographic inaccessibility, relies substantially on a junior clinical workforce posted to remote district facilities, conditions that may intensify interpersonal occupational stress and restrict access to structured professional development (14,15). Rajasthan, one of India's largest states by area, is characterised by significant rural-urban disparities in healthcare access, a provider-to-population ratio below the national average in many districts, and a predominantly public-sector delivery model that places considerable strain on facility-based clinical teams (16).Gujarat, an economically developed western state with a relatively dense tertiary care infrastructure and substantial private sector participation, offers a contrasting organisational context in which professional norms, training culture, and institutional hierarchies may differ substantively from public sector facilities in less-developed states (17). Telangana, formed in 2014 following bifurcation from Andhra Pradesh, has rapidly expanded its public health workforce through several state-level health assurance programmes; however, training infrastructure for interprofessional competencies in this nascent system remains limited (18).

### 2.3 Study population

The target population comprised practising healthcare professionals, specifically qualified doctors (medical officers and specialists), registered nurses, and interns (undergraduate medical students in compulsory rotating internships and nursing interns in supervised clinical placements), who were employed or posted at the participating facilities during the data collection period. Individuals were eligible for inclusion if they met all of the following criteria: formal employment or institutional attachment in a clinical (patient-facing) capacity; at least one month of continuous direct patient care experience; and provision of written informed consent. Participants were excluded if they were on extended leave (medical, administrative, or otherwise) at the time of recruitment, occupied exclusively non-clinical or managerial roles, or declined to participate.

### 2.4 Sample size

The minimum required sample size was calculated using the standard formula for estimating a single proportion: *n* = Z²p(1−p)/d², where Z = 1.96 (two-tailed, 95% confidence level) (19), *p* = 0.50 (there is no published national cross-sectional study reporting the proportion of Indian healthcare workers who perceive workplace conflict.), and d = 0.05 (acceptable absolute precision). This yielded a minimum of 384 participants. Accounting for an anticipated non-response rate of 10% and applying a design effect of 1.5 to correct for intracluster correlation inherent in the multi-stage sampling design, the target sample was inflated to approximately 633 participants. A final analytical sample of 566 healthcare professionals was retained following the exclusion of questionnaires with incomplete responses to any of the primary variables (retention rate: 89%).

### 2.5 Sampling strategy

A multi-stage stratified sampling design was employed. In the first stage, each of the four participating states constituted a sampling stratum; within each stratum, one or two health facilities were purposively selected on the basis of institutional accessibility, case load representativeness, and administrative approval. In the second stage, eligible participants within each selected facility were recruited from clinical departments using systematic random sampling: individuals were selected from departmental staff rosters at predetermined fixed intervals. Proportional allocation across professional categories (doctor, nurse, intern) was planned; however, given the differential availability of cadres at the time of data collection, the final sample reflects a convenience-stratified composition.

### 2.6 Data collection instrument

Data were collected using a structured, self-administered questionnaire developed through a three-phase process. In phase one, survey items were generated through an extensive review of validated instruments used in interprofessional conflict and workplace relations research, including the Nurse-Physician Collaboration Scale (20) and the Workplace Conflict Assessment Tool (21). In phase two, the draft instrument was submitted to a panel of five content experts, two public health physicians, two nursing faculty members, and one biostatistician, for iterative assessment of content validity, relevance, and item clarity; revisions were made until consensus was reached. In phase three, a pilot study was administered to 30 healthcare professionals who were subsequently excluded from the main analysis; this phase confirmed item comprehension and acceptable response burden, and yielded satisfactory internal consistency for the conflict consequences subscale (Cronbach's *α* = 0.82). The finalised questionnaire comprised two sections: (i) sociodemographic and professional characteristics (age, gender, role, years of experience); and (ii) inter-professional conflict scale. The scale consists of 22 items (Table 5). Analysis for 12 items rated on a five-point Likert scale (1 = Strongly Disagree, 5 = Strongly Agree), covering perceived conflict prevalence (Conflict is common in workplace, Personal experience with conflict) consequences for patient care (Conflict affects patient care quality, Conflict delays patient care, Conflict impacts patient outcomes, Conflict causes medical errors) and team functioning(Conflict strains team relationships, Conflict decreases collaboration, Conflict increases stress), training adequacy (Received adequate conflict training, Comfortable addressing conflict) and receptivity to training (Believe training would help) are presented in this manuscript.

### 2.7 Variables

#### 2.7.1 Outcome variables

The primary outcome variable was the perceived conflict frequency in the workplace. Secondary outcome variables included self-reported adequacy of received conflict management training, operationalised as the mean score on the item “I have received adequate training in conflict management” (5-point Likert scale), perceived consequences for patient care quality and team collaboration, and receptivity to conflict management training. The training adequacy gap was operationally defined as the arithmetic difference between the mean score for the item “Training in conflict management would help resolve workplace conflict” and the mean score for training adequacy.

#### 2.7.2 Independent variables

Professional role (doctor, nurse, or intern), gender (male or female), age (years, continuous), and professional experience (years, categorised as ≤5, 6–10, 11–20, and >20) were treated as the primary independent variables. Experience was additionally treated as a binary variable (≤5 years versus >5 years) in sensitivity analyses, consistent with practice in health workforce research.

### 2.8 Statistical analysis

All analyses were performed using IBM SPSS Statistics version 26.0 (IBM Corporation, Armonk, NY, USA). Categorical variables are described as absolute frequencies and percentages; continuous and ordinal variables are described as means with standard deviations (SD) and 95% confidence intervals (CI). For each Likert item, the complete distribution of responses across all five response categories is additionally reported as percentages.

For the primary training gap analysis, the mean scores of the training adequacy and training importance items were each compared against the theoretical scale midpoint (3.0) using a one-sample t-test. The inter-item difference was calculated, and the practical magnitude of the gap was quantified using Cohen's d, with values of 0.20, 0.50, and 0.80 defined *a priori* as thresholds for small, medium, and large effects, respectively (22).

For subgroup analysis by professional role, one-way analysis of variance (ANOVA) was used to compare mean scores across the three professional categories for each of the 12 survey items. Where significant omnibus F-statistics were obtained (*p* < 0.05), Tukey's honestly significant difference (HSD) *post-hoc* test was applied to identify pairwise differences. Effect sizes for role differences were estimated using partial eta-squared (*η*²), with thresholds of 0.01, 0.06, and 0.14 corresponding to small, medium, and large effects, respectively. Given that Likert scale data represent ordinal rather than interval measurement, Spearman's rank-order correlation coefficient (*ρ*) was used to characterise bivariate associations among all 12 survey items. The alpha level for all inferential tests was set at 0.05 (two-tailed). Missing data were minimal (< 1% across all items) and handled using listwise deletion; sensitivity analyses confirmed that results were not materially affected by this approach.

### 2.9 Ethical considerations

The study protocol was reviewed and approved by the Institutional Ethics Committee of the coordinating institution prior to commencement of data collection (Tomo Riba Institute of Health and Medical Sciences (TRIHMS), Reference Number: TRIHMS/IEC/2023/3242-3245;ESIC MCH Hyderabad, Telangana, Reference Number: 799/U/IEC/MC/F552/11/2023; Geetanjalai University, Udaipur, Reference Number: GU/HREC/EC/2022/2165, Shantabaa Medical College SMC/IEC/GO/06/24.). All participation was voluntary. Written informed consent was obtained from each eligible participant before questionnaire administration, and participants were explicitly informed of their right to withdraw at any stage without consequence to their employment status or professional standing. Questionnaire forms were anonymous and contained no personal identifiers; completed forms were stored in a secure, locked repository. Digital data were maintained in a password-protected repository accessible only to the principal investigators. All procedures complied with the Declaration of Helsinki (revised 2013) and applicable national ethical guidelines for health research involving human participants.

## 3 Results

### 3.1 Participant characteristics

A total of 566 healthcare professionals were enrolled in the study. Gender distribution was near-balanced, with 51.8% female and 47.3% male respondents. Doctors constituted the largest professional subgroup (52.7%), followed by nurses (35.7%) and interns (11.6%). The cohort was predominantly junior: the mean age was 29.8 years (SD = 7.6) and the median years of professional experience was 3.0 (IQR = 6.0). Nearly 70% of participants reported five or fewer years of clinical experience (Table 1), suggesting that the sample broadly reflects the perspectives of early-career healthcare workers. No missing data were recorded for the primary demographic variables.

**Table 1:** Demographic and professional characteristics of study participants (*N* = 566).

Characteristic	Category	n	%
Gender	Male	268	47.3
	Female	298	51.8
	Missing/Not specified	—	0.9
Professional Role	Doctor	298	52.7
	Nurse	202	35.7
	Intern	66	11.6
Age (years)	Mean (SD)	29.8 (7.6)	
	Median (IQR)	28.0 (8.0)	
	Range	21–67	
Experience (years)	Mean (SD)	5.5 (6.6)	
	Median (IQR)	3.0 (6.0)	
Experience categories	≤5 years	395	69.8
	6–10 years	91	16.1
	11–20 years	59	10.4
	>20 years	21	3.7
State	Gujarat (1 hospital)	77	14
	Rajasthan (1 hospital)	82	14
	Arunachal Pradesh (1 hospital)	135	24
	Telangana (2 hospitals)	272	48

IQR, interquartile range; SD, standard deviation.

### 3.2 Perceived prevalence and effect of interprofessional conflict

Descriptive statistics for all 12 Likert-scaled survey items are presented in Table 2. Conflict was widely perceived as a frequent occurrence in the workplace, with a mean score for the item *Conflict is common in my workplace* approaching “Agree” on the 5-point scale (95% CI: 3.62–3.79). More than 60% of respondents agreed or strongly agreed with this statement. The perceived effect on patient care quality was similarly prominent (95% CI: 3.50–3.71), with over 60% of respondents endorsing agreement. Conflict-related stress emerged as a substantive secondary concern, with approximately 56% of participants agreeing that interprofessional conflict contributed to increased personal stress.

**Table 2:** Descriptive statistics of key study variables (*N* = 566).

Variable	n	Mean	SD	95% CI	SD%	Agr%	SA%
Conflict is common in workplace	566	3.70	1.06	3.62–3.79	14.3	37.5	25.3
Conflict affects patient care quality	566	3.60	1.23	3.50–3.71	18.5	32.7	27.6
Comfortable addressing conflict	566	3.23	1.28	3.12–3.33	30.7	25.6	19.4
Received adequate conflict training	566	2.95	1.33	2.84–3.06	36.2	23.3	14.1
Personal experience with conflict	566	3.25	1.23	3.15–3.35	26.3	32.3	15.5
Conflict strains team relationships	566	3.32	1.12	3.22–3.41	22.1	33.7	14.0
Conflict delays patient care	566	3.07	1.22	2.97–3.17	29.5	23.7	13.4
Conflict decreases collaboration	566	3.32	1.12	3.23–3.41	23.8	35.9	13.6
Conflict impacts patient outcomes	566	3.09	1.21	2.99–3.19	30.9	29.0	11.8
Conflict increases stress	566	3.51	1.15	3.42–3.61	18.0	34.5	21.2
Conflict causes medical errors	565	3.09	1.23	2.99–3.19	30.8	23.9	14.3
Believe training would help	566	3.88	1.01	3.80–3.96	7.5	39.6	29.9

SD% = combined Strongly Disagree + Disagree percentage; Agr% = Agree percentage; SA% = Strongly Agree percentage. CI, confidence interval; SD, standard deviation. Items rated on a 5-point Likert scale (1 = Strongly Disagree, 5 = Strongly Agree).

**Figure 1:**
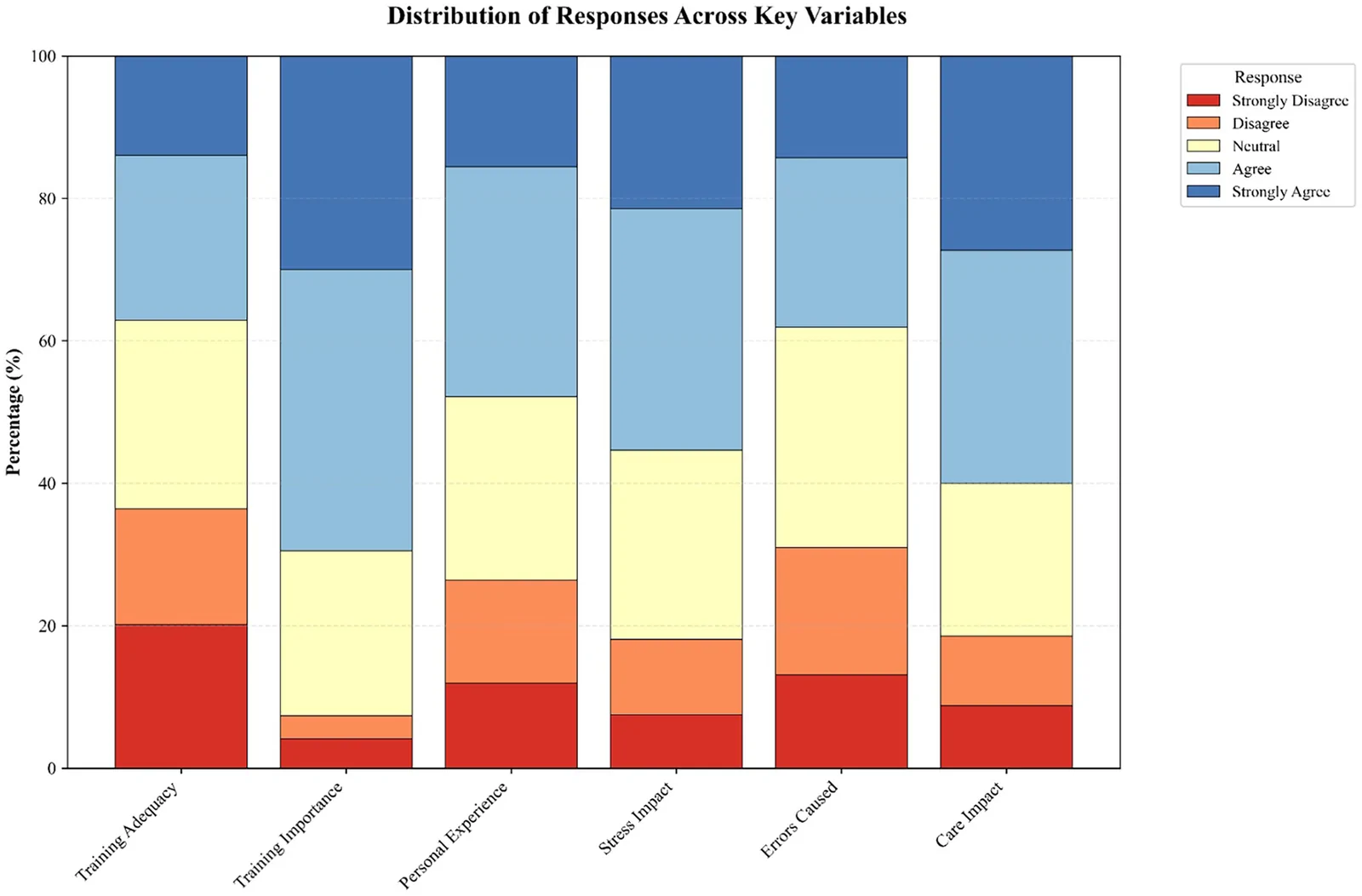
Distribution of responses across key variables.

The item with the highest endorsement across the sample was *Believe training would help* (95% CI: 3.80–3.96), with nearly 70% of participants expressing agreement, including 29.9% indicating strong agreement. Conversely, *Received adequate conflict management training* recorded the lowest mean score (95% CI: 2.84–3.06) and the highest proportion of combined disagreement. The response distribution pattern across key items is visualised in Figure 1, which illustrates a systematic skew toward agreement for conflict-impact items alongside a contrasting concentration of disagreement for training adequacy — a pattern consistent with a training deficit in the studied population.

### 3.3 Training adequacy gap

Paired comparison of current training adequacy and perceived training importance revealed a clinically and statistically meaningful deficit. The mean score for training adequacy fell below the scale midpoint (3.0), whereas perceived training importance approached 4.0 (Table 3). A one-sample comparison against the neutral midpoint (3.0) confirmed that both measures differed significantly from chance (both *p* < 0.001). The inter-item difference of 0.93 Likert points corresponded to a large effect size (Cohen's d = 0.78), indicating that this gap represents not merely a statistical artefact but a substantively important finding with practical relevance for curriculum development and institutional policy.

**Table 3:** Training gap analysis: current adequacy vs. perceived Importance of conflict management training.

Measure	Mean (SD)	Comparison vs. Midpoint (3.0)	*p*-value	Cohen's d
Current Training Adequacy	2.95 (1.33)	Below midpoint (↓)	<0.001	0.78
Perceived Training Importance	3.88 (1.01)	Well above midpoint (↑)	<0.001	Large
Mean Difference	**0.93 points**	Near 1-point gap on a 5-point Likert scale	**<0.001**	**0.78 (large)**

Cohen's d calculated relative to scale midpoint (3.0). Large effect defined as d ≥ 0.80 (Cohen, 1988). *p*-values derived from one-sample t-test against midpoint.

Bold values highlight the key outcome of the analysis (training gap), including the mean difference and corresponding large effect size (Cohen's d = 0.78 ) indicates a large practical effect size, suggesting a substantial training need.

### 3.4 Subgroup analysis: conflict perceptions by professional role

**Figure 2:**
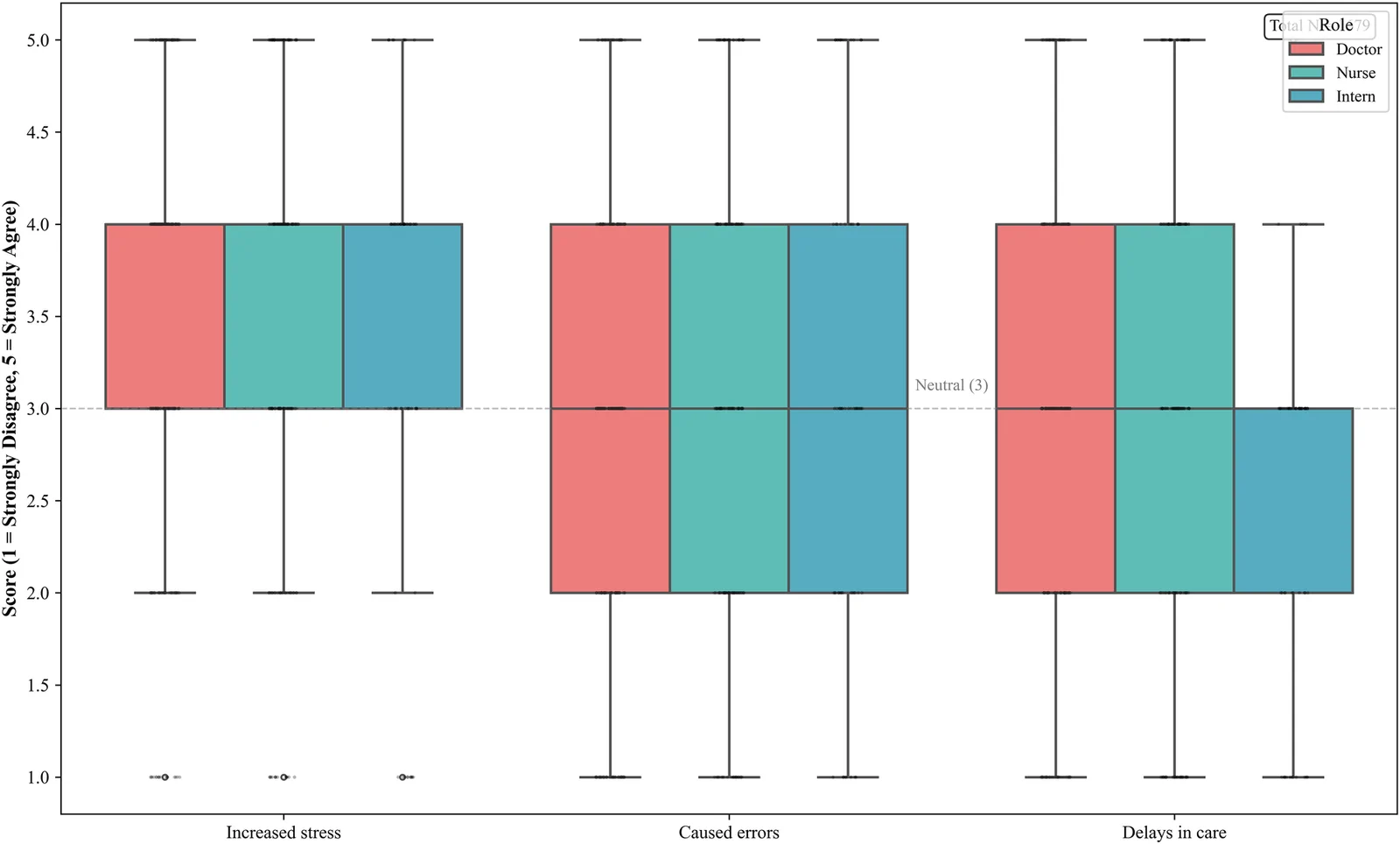
Perceived impact of conflict by professional role.

**Figure 3:**
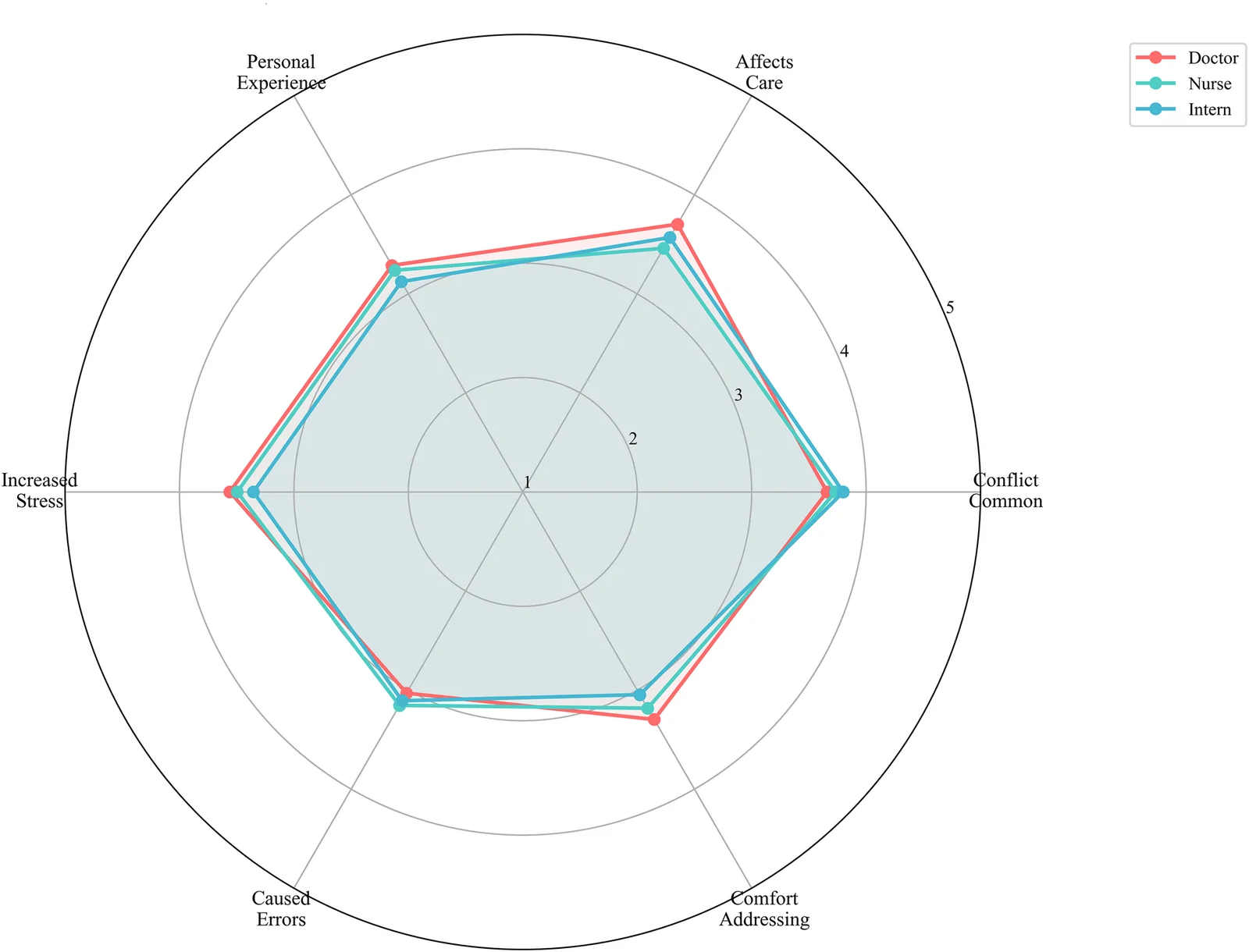
Radar chart comparing doctors, nurses, and interns on six key variables.

One-way ANOVA was conducted to examine whether professional role (doctor, nurse, intern) was associated with differences in conflict-related perceptions across the 12 survey domains. Results are presented in Table 4 and visualised in Figures 2, 3. For the majority of conflict-related dimensions — including frequency, patient care impact, team relationship strain, decreased collaboration, medical error attribution, and perceived benefit of training — no statistically significant inter-role differences were identified (all *p* > 0.05, all effect sizes *η*² < 0.01), suggesting a broadly shared perception of conflict across hierarchical levels.

**Table 4:** One-way ANOVA: comparison of conflict-related variables by professional role.

Variable	Dr Mean	Dr SD	Nu Mean	Nu SD	In Mean	In SD	F	*p*-value
Conflict is common in workplace	3.66	0.99	3.73	1.17	3.80	1.05	0.60	0.548
Conflict affects patient care quality	3.70	1.16	3.46	1.30	3.57	1.27	2.37	0.095
Comfortable addressing conflict	3.30	1.20	3.18	1.35	3.05	1.34	1.22	0.296
Received adequate conflict training	2.90	1.31	3.19	1.31	2.37	1.26	9.97	<0.001*
Personal experience with conflict	3.29	1.22	3.24	1.27	3.12	1.14	0.49	0.615
Conflict strains team relationships	3.38	1.10	3.27	1.13	3.15	1.19	1.27	0.281
Conflict delays patient care	3.12	1.22	3.11	1.25	2.72	1.07	2.97	0.052
Conflict decreases collaboration	3.38	1.08	3.31	1.17	3.14	1.14	1.20	0.301
Conflict impacts patient outcomes	3.07	1.22	3.17	1.19	2.92	1.20	1.09	0.337
Conflict increases stress	3.26	1.15	3.65	1.16	3.50	1.22	6.84	0.001*
Conflict causes medical errors	3.03	1.22	3.15	1.21	3.11	1.30	0.62	0.540
Believe training would help	3.87	1.07	3.88	0.90	3.95	1.07	0.20	0.819

Dr = Doctor; Nu = Nurse; In = Intern. Significant *p*-values (*p* < 0.05) are bolded and marked with an asterisk (*). *η*² effect sizes: 0.01 = small, 0.06 = medium, 0.14 = large. *post-hoc* pairwise comparisons not shown; available upon request.

Two variables demonstrated statistically significant between-group differences. First, perceived adequacy of received conflict management training differed significantly by role (F = 9.97, *p* < 0.001, *η*² = 0.035), with nurses reporting higher adequacy than interns. *post-hoc* interpretation suggests that interns may be particularly under-served by existing training provisions. Second, the perception that conflict increases personal stress differed by professional group (F = 6.84, *p* = 0.001, *η*² = 0.030), with nurses reporting the highest stress burden relative to doctors. As shown in Figure 2, the interquartile ranges for these variables were wide across all roles, indicating considerable within-group heterogeneity. Figure 3 (radar chart) further confirms that while all three professional groups display similar multidimensional conflict profiles, nurses appear to score modestly higher on stress-related and care-impact dimensions, whereas doctors report marginally greater comfort addressing conflict directly.

### 3.5 Correlation structure of conflict-related variables

**Figure 4:**
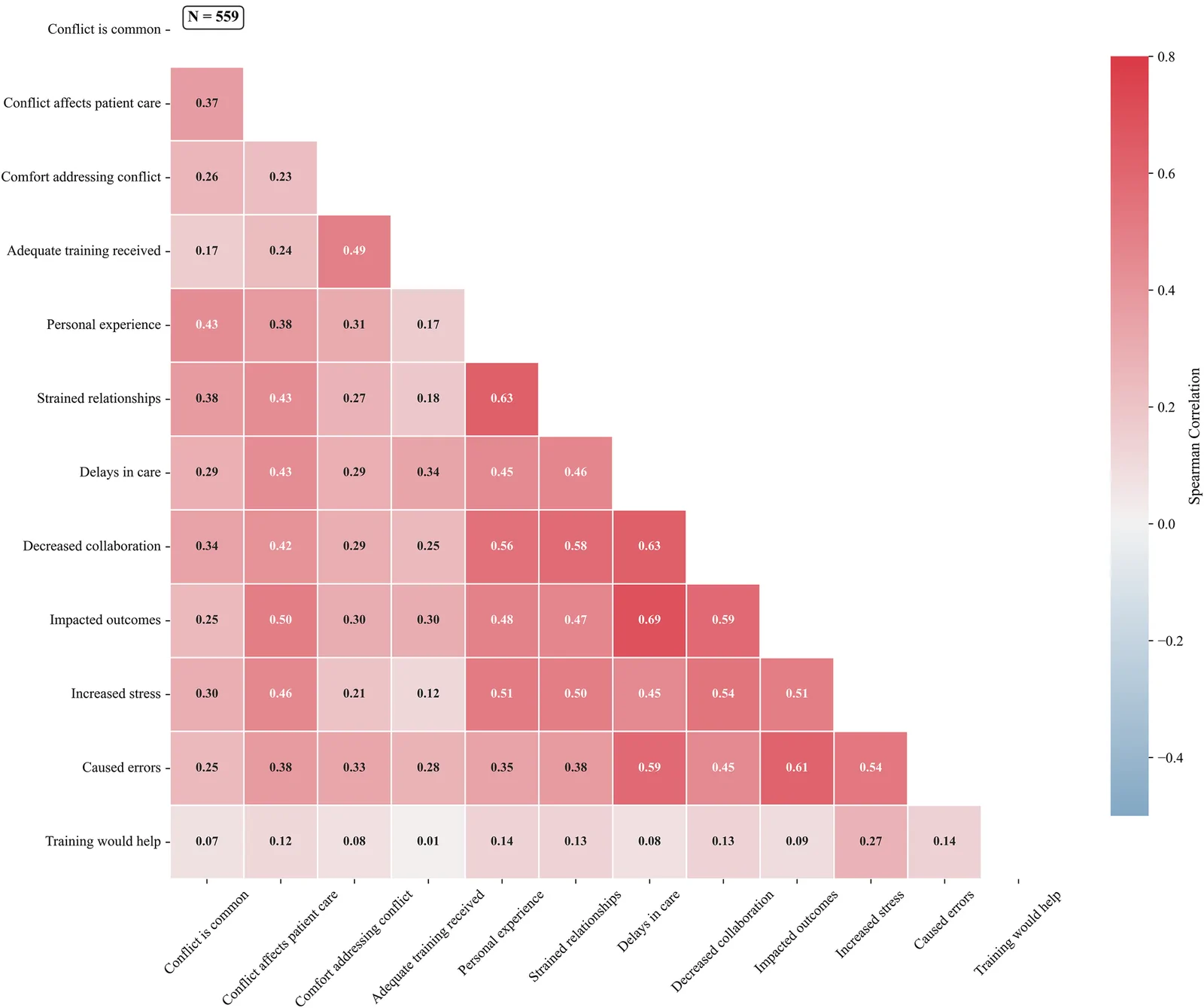
Correlation matrix of conflict-related variables in healthcare teams.

Spearman rank-order correlations were computed among all 12 conflict-related variables, and are presented in the lower triangle of the correlation matrix (Figure 4). The correlation matrix revealed a coherent and theoretically expected pattern of positive inter-item associations. The strongest correlation observed was between *conflict decreases collaboration* and *conflict delays patient care* (*ρ* = 0.63, *p* < 0.001), indicating that respondents who perceived interprofessional conflict as disrupting collaborative function also recognised downstream consequences for care timeliness. A similarly strong association was found between *personal experience with conflict* and *conflict strains team relationships* (*ρ* = 0.63, *p* < 0.001), suggesting that direct conflict exposure is tightly coupled with perceived relational damage within the team.

Moderate correlations (*ρ* range: 0.43–0.56) were broadly observed between impact-related items (stress, errors, delays, outcomes, and decreased collaboration), indicating substantial shared variance across negative conflict sequelae. Importantly, *conflict impacts patient outcomes* was strongly associated with both *conflict causes medical errors* (*ρ* = 0.61, *p* < 0.001) and *conflict decreases collaboration* (*ρ* = 0.59, *p* < 0.001). The belief that training would help exhibited the weakest correlations across all items (*ρ* range: 0.07–0.27), with its strongest association being with *conflict increases stress* (*ρ* = 0.27, *p* < 0.001). This pattern, visible in the rightmost column of Figure 4, suggests that motivation for training may be partially driven by personal stress experience rather than abstract awareness of conflict consequences.

### 3.6 Approaches for reducing interpersonal conflict

Overall, the respondents demonstrated positive attitudes toward all proposed strategies for managing interpersonal conflict, with mean scores approaching 4.0. The highest levels of agreement were observed for interventions emphasizing respectful workplace culture, effective communication, and active listening, suggesting that healthcare professionals consider these interpersonal and organizational factors more influential than training alone. The findings imply that successful conflict management in healthcare settings requires a multifaceted approach combining organizational culture, structured communication, leadership engagement, standardized protocols, and continuous professional development (Table 5).

**Table 5:** One-way ANOVA: comparison of all items of the scale by professional role.

Themes	Variable	Dr Mean	Dr SD	Nu Mean	Nu SD	In Mean	In SD	F	p-value	Agree (All cadre)	Mean	SD
Perceptions of Conflict in Healthcare Teams	1. Conflict is a common occurrence in within healthcare teams.	3.66	0.989	3.73	1.171	3.8	1.05	0.592	0.554	63%	3.7	1.06
	2. Conflict within healthcare teams negatively affects team dynamics.	3.79	1.132	3.58	1.28	3.88	1.06	2.294	0.104	68%	3.72	1.18
	3. Conflict within healthcare teams hinders effective communication.	3.81	1.053	3.63	1.207	3.88	1.222	1.772	0.173	68%	3.76	1.13
	4. Conflict within healthcare teams affects the quality of patient care.	3.71	1.166	3.46	1.305	3.58	1.266	2.39	0.095	60%	3.6	1.23
	5. Healthcare staff members feel comfortable addressing conflicts within their teams.	3.3	1.207	3.18	1.35	3.03	1.336	1.334	0.296	45%	3.23	1.28
	6. Conflict within healthcare teams leads to decreased job satisfaction.	3.66	1.215	3.36	1.379	3.59	1.252	3.239	0.042	59%	3.54	1.29
	7. Conflict within healthcare teams affects the overall morale of the team.	3.8	1.077	3.46	1.301	3.83	1.171	5.141	0.007	64%	3.68	1.18
	8. Healthcare staff members are adequately trained in conflict resolution strategies.	2.92	1.317	3.19	1.314	2.39	1.263	9.773	<0.001*	37%	2.95	1.33
Experiences of Conflict in Healthcare Teams	1. I have personally experienced conflict within my healthcare team.	3.29	1.216	3.24	1.271	3.14	1.135	0.488	0.615	48%	3.25	1.23
	2. Conflict within my healthcare team has resulted in strained relationships.	3.39	1.099	3.27	1.132	3.15	1.19	1.27	0.281	48%	3.32	1.12
	3. Conflict within my healthcare team has caused delays in patient care.	3.12	1.221	3.11	1.255	2.71	1.064	2.97	0.052	37%	3.07	1.22
	4. Conflict within my healthcare team has led to decreased collaboration.	3.38	1.081	3.31	1.175	3.14	1.135	1.13	0.301	49%	3.32	1.12
	5. I believe that conflicts within my healthcare team have impacted patient outcomes.	3.07	1.215	3.17	1.193	2.91	1.199	1.09	0.337	41%	3.09	1.21
	6. Conflict within my healthcare team has increased stress levels among team members.	3.26	1.15	3.65	1.16	3.5	1.22	6.84	0.001*	56%	3.51	1.15
	7. Conflict within my healthcare team has caused errors or mistakes in patient care	3.04	1.221	3.15	1.21	3.12	1.295	0.62	0.54	38%	3.09	1.22
Solutions for Managing Conflict in Healthcare Teams	1. Adequate training in conflict resolution can improve team dynamics within healthcare teams.	3.87	1.07	3.88	0.9	3.95	1.07	0.2	0.819	69%	3.88	1.01
	2. Implementing clear communication channels can help prevent conflicts within healthcare teams.	3.95	1.08	4.02	0.875	4	1.081	0.324	0.724	74%	3.98	1.01
	3. Regular team-building activities can improve relationships and decrease conflicts in healthcare teams.	3.96	1.018	3.95	0.924	4.03	1.109	0.141	0.869	72%	3.96	0.995
	4. Establishing standardized protocols for conflict resolution can effectively manage conflicts within healthcare teams.	3.87	1.085	4.01	0.962	3.97	1.24	1.181	0.309	72%	3.93	1.06
	5. Encouraging open dialogue and active listening can help address conflicts within healthcare teams.	3.92	1.037	3.98	0.954	4.33	1.043	4.211	0.016	72%	3.99	1.01
	6. Leadership support and involvement are crucial in resolving conflicts within healthcare teams.	3.88	1.087	3.95	0.934	4.03	1.265	0.529	0.59	70%	3.92	1.06
	7. Developing a culture of respect and empathy can prevent conflicts and improve patient care within healthcare teams.	3.96	1.047	4.01	0.936	4.12	1.183	0.568	0.568	74%	4	1.03

Dr = Doctor; Nu = Nurse; In = Intern. Significant *p*-values (*p* < 0.05) are bolded and marked with an asterisk (*). *η*² effect sizes: 0.01 = small, 0.06 = medium, 0.14 = large. *post-hoc* pairwise comparisons not shown; available upon request.

## 4 Discussion

### 4.1 Principal findings

This multi-state cross-sectional study of healthcare professionals across four geographically and contextually distinct Indian states found that interprofessional conflict is widely perceived as common within healthcare settings. Existing conflict-management training provisions are viewed as in sufficient the substantial difference observed between perceived training adequacy and the perceived importance of such training (0.93 Likert points; Cohen's d = 0.78) suggests a notable unmet educational need among healthcare professionals.

The study also identified moderate to strong positive correlations between perceptions of interprofessional conflict and perceived adverse consequences for teamwork, workplace stress, and patient care processes. However, given the cross-sectional design and reliance on self-reported perceptions, these associations should not be interpreted as evidence of causal relationships. Alternative explanations, including shared workplace environments, organizational culture, workload pressures, and reporting tendencies, may have contributed to the observed patterns. Available literature suggests that conflict, stress, and training gaps are shaped by hierarchical position in clinical settings, with junior cadres such as interns and nurses often appearing more vulnerable to inadequate preparation and workplace stress than senior doctors (23, 24).

### 4.2 Comparison with existing literature

The high prevalence of perceived interprofessional conflict documented in this study, affirmed by over 60% of respondents across all sites, is broadly consistent with findings from prior Indian and South Asian studies despite the methodological differences. A hospital-based study from south India further identified conflict as a significant contributor to nursing staff attrition, with junior nurses disproportionately affected — a pattern corroborated by the significantly elevated stress scores among nurses observed in the present data (F = 6.84, *p* = 0.001) (25). In a study among nurses working in New Delhi, 45.1% agreed that there were clashes between subgroups within their group (26). In a study conducted among 58 health healthcare professionals in four healthcare settings in the southeast of Nigeria, 80% reported some form of interprofessional conflict (27). A total of 76.6% of doctors and 96.7% of nurses reported that they had experienced at least one interprofessional conflict while working at the hospital at Seol, South Korea (28). Similar such findings have been reported world wide (29, 30). Internationally, Roche et al. documented that over 70% of hospital nurses in Australia had experienced workplace conflict in the preceding month (31) while a European multisite study by Arenas et al. similarly found that conflict-related psychological burden disproportionately affected nursing staff relative to physicians, a pattern replicated in the present Indian cohort and suggestive of a nursing-specific vulnerability to conflict-related occupational stress that may transcend healthcare system boundaries (32).

The training adequacy gap identified in this study is consistent with patterns documented in multiple national and international contexts. Similar reactions were received in a national survey of Indian critical care physicians who perceived insufficient formal communication training during residency (33). Internationally, McKinley et al. documented that fewer than 30% of medical graduates in the United Kingdom felt adequately equipped to navigate interprofessional conflict in clinical settings (34), and a systematic review by Abramson and Mizrahi concluded that conflict management remained among the least formally addressed competencies across health professions education programmes globally (35).

The observed correlation between conflict exposure and perceived patient harm, with Spearman coefficients ranging from *ρ* = 0.43 to 0.69 among consequence-related variable pairs, aligns with the organisational safety literature. Rosenstein and O'Daniel's well-cited framework describing the “conflict-to-care” pathway, in which disruptive clinical behaviour and unresolved interprofessional tension are associated with medication errors and near-miss events, provides theoretical grounding for the present findings (36).

### 4.3 Policy and educational implications

The training adequacy gap identified in this study, substantial in magnitude, consistent across professional roles and experience categories, and accompanied by near-universal willingness to receive training, provides quantitative justification for prioritising conflict management within health professional education and institutional development programmes. The National Medical Commission (NMC) and the Indian Nursing Council are well-positioned to incorporate structured conflict resolution and interprofessional communication competencies into revised curriculum frameworks for undergraduate and postgraduate healthcare programmes, consistent with the interprofessional education principles articulated in the WHO's 2010 Framework for Action on Interprofessional Education and Collaborative Practice (37). The National Medical Commission as the primary vehicle for in-service workforce development, should consider integrating conflict management modules into existing induction, orientation, and continuing professional development programmes, with particular attention to internship rotations and junior nursing induction, given the significantly lower training adequacy reported by interns (F = 9.97, *p* < 0.001) and the elevated stress burden among nurses.

The observation that receptivity to training was most strongly correlated with personal experience of conflict-related stress (*ρ* = 0.27, *p* < 0.001) has implications for programme design and communication. Framing conflict management training as an occupational health and burnout prevention initiative, rather than exclusively as a governance or professionalism requirement, may be more effective in driving voluntary uptake, particularly among nursing staff who bear the greatest stress burden in this sample (38).

These findings suggest that healthcare professionals perceive conflict management as a shared organizational responsibility rather than solely an individual competency. The highest-rated interventions—developing a culture of respect and empathy, promoting open dialogue, and strengthening communication channels—underscore the importance of psychologically safe work environments where team members can express concerns without fear of blame. Collectively, these results support the implementation of comprehensive conflict-management programs that combine communication training, leadership development, team-building initiatives, and formal conflict-resolution mechanisms to strengthen interprofessional collaboration and ultimately improve the quality of patient care.

### 4.4 Strengths

This study offers several design and analytical strengths. The multi-state sampling framework, spanning four geographically and contextually diverse states, represents a substantive advance over single-institution studies and allows a degree of cross-contextual inference that single-site designs cannot support. The inclusion of three groups of health care professionals within a single analytical framework enables examination of hierarchical dynamics that are obscured in cadre-specific studies. The study employed a structured questionnaire developed through literature review, expert assessment, and pilot testing, demonstrating acceptable internal consistency for the conflict-consequences subscale. Rigorous statistical reporting, including exact *p*-values, 95% confidence intervals, and effect sizes benchmarked to established thresholds (Cohen's d, *η*²), ensures methodological transparency and facilitates comparison with the existing international literature. The high questionnaire completion rate (89%) reduces the risk that non-response bias materially affected the findings. The multi-method visual analytic strategy, integrating correlation heatmaps, boxplots, stacked bar charts, and radar charts, communicates multidimensional findings at multiple levels of granularity and enhances accessibility for both specialist and policy audiences. The study addresses an important evidence gap regarding interprofessional conflict and training needs within the Indian healthcare context and provides data that may inform future policy, educational interventions, and research initiatives.

### 4.5 Limitations

Several limitations should be considered when interpreting the findings of this study. First, the cross-sectional design precludes causal inference; therefore, the observed associations between conflict perceptions, workplace outcomes, and training-related variables cannot establish temporal or causal relationships. Second, all measures were based on self-reported perceptions, making the findings susceptible to recall bias, social desirability bias, and subjective interpretation. The study assessed perceived impacts on patient care and teamwork rather than objectively measured patient safety outcomes. Third, although a multi-stage sampling framework was employed, participating institutions were selected purposively and practical constraints resulted in some convenience elements during participant recruitment. Consequently, the sample may not be fully representative of healthcare professionals across all Indian healthcare settings.

Fourth, the study did not distinguish between different types of conflict, such as interpersonal, professional, organizational, or structural conflict. As a result, the findings provide limited insight into the specific mechanisms through which different forms of conflict may influence workplace experiences and patient care.Fifth, potentially important contextual and organizational variables—including workload, staffing adequacy, department type, leadership style, institutional culture, and previous exposure to conflict-management training—were not measured. The absence of these variables limited adjustment for potential confounding factors and restricted exploration of heterogeneity across settings and professional groups.

Sixth, although the questionnaire underwent expert review, pilot testing, and demonstrated acceptable internal consistency, comprehensive psychometric validation, including construct validation and confirmatory factor analysis, was not performed in the study population. Future studies should undertake more extensive validation procedures prior to large-scale implementation. Finally, the analytical approach focused primarily on descriptive, comparative, and bivariate analyses. Multivariable modelling was beyond the scope of the present study and therefore independent predictors of conflict perceptions and training adequacy could not be identified. Future longitudinal and mixed-methods studies incorporating multivariate analyses would provide deeper understanding of causal pathways, contextual influences, and subgroup-specific experiences.

## 5 Conclusion

Healthcare professionals perceived interpersonal conflict to be common and detrimental within healthcare settings. Respondents reported limited comfort in addressing interpersonal conflict, suggesting deficiencies in conflict-management culture and psychological safety. Personal experience of interpersonal conflict was frequently reported across professional groups. Occupational stress and strained professional relationships emerged as the most commonly perceived consequences of interpersonal conflict. The perceived effect of interpersonal conflict on patient care and clinical errors was moderate compared with its impact on staff well-being and teamwork. Respondents expressed strong support for system-level interventions—including leadership engagement, structured communication, conflict-resolution training, and fostering a culture of respect and empathy—as effective strategies for preventing and managing interpersonal conflict. The study urges making structured, cadre-sensitive interprofessional conflict-management training a top systems-level priority to strengthen teamwork, workforce well-being, and patient outcomes in India's healthcare system. National healthcare accreditation standards should include indicators related to workplace culture, psychological safety, teamwork, and conflict management. Healthcare institutions should routinely assess workplace conflict and staff well-being through periodic organizational climate surveys. Integrating stress management, employee assistance programs, counselling services, and wellness initiatives will help reduce the psychological burden associated with workplace conflict

## Data Availability

The raw data supporting the conclusions of this article will be made available by the authors, without undue reservation.
